# A Half-Century of Inspiration: An Interview with Hamilton Smith

**DOI:** 10.1371/journal.pgen.1002466

**Published:** 2012-01-12

**Authors:** Jane Gitschier

**Affiliations:** Department of Medicine and Pediatrics, University of California San Francisco, San Francisco, California, United States of America

In 1962, Hamilton Smith abandoned a career in medicine to follow his passion for the emerging field of molecular biology; within six years, he had made the discovery of a lifetime. As a new Johns Hopkins faculty member, Smith, together with his first graduate student, Kent Wilcox, geared up to study recombination in vitro but instead discovered the restriction enzyme “R” in *Haemophilus influenzae*. By cobbling together crude techniques, Smith, along with Wilcox and later Tom Kelly, showed that R cleaves DNA at a specific recognition sequence, a palindromic site, yielding blunt-ended DNA fragments. Now known as HindII, R proved to be the first of an enormous class of Type II restriction enzymes, and as such, presaged gene cloning, allowed DNA to be reproducibly fragmented and then sequenced, and enabled physical mapping of genomes. Smith went on to discover DNA methylases that constitute the other half of the bacterial host restriction and modification systems, as hypothesized by Werner Arber of Switzerland. Together with Arber and his Hopkins colleague Daniel Nathans, who first used the enzyme on SV40 DNA and demonstrated discrete bands on a tube gel, Smith shared the Nobel Prize for Physiology or Medicine in 1978.

Smith's curiosity and his gift for hands-on research continued to guide him through a highly productive career at Hopkins for more than three decades, when a chance meeting with Craig Venter, who had just launched The Institute for Genome Research (TIGR), turned his attention to sequencing the *Haemophilus* genome. In 1998, he gave up his faculty position at Hopkins and has been working with Venter ever since. Currently, Smith (Image 1) spearheads the highly visible synthetic biology program at the J. Craig Venter Institute (JCVI) in San Diego. And that is where I caught up with him in late October.

**Figure 1:**
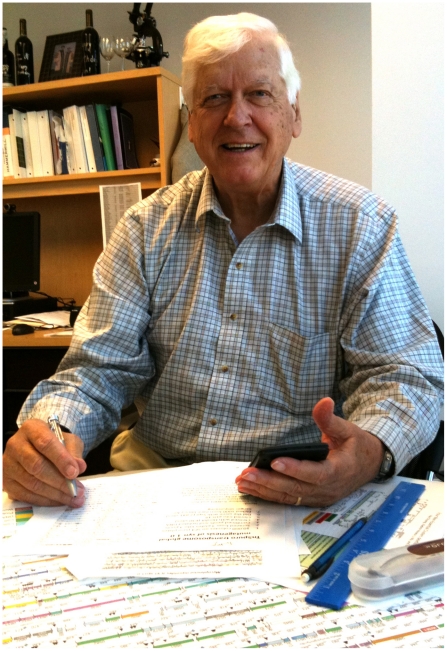
Hamilton Smith.

I discovered a soft-spoken and cheerful man whose focus and energy have buoyed him through a half-century of science and who shows no signs of wearing down. We pick up the conversation where Ham gets the idea of going into research.

**Gitschier:** Let's start with how you got involved in basic research after going to medical school.

**Smith:** After my internship [at Barnes Hospital in Saint Louis], the plan was just to go on with the system—residency. At that time there was no draft, but there was a doctors' draft. There weren't enough doctors volunteering to serve, so by lottery they would select one out of every ten physicians for a two-year period. And my number came up. I was just married, so we headed off to San Diego for two years in the Navy.

I was head of the dispensary at the 11th Naval District Headquarters, down on Pacific Highway. I had about seven or eight corpsmen assisting me. I was the doctor taking care of about 1,100 civilians and 500–600 military, mostly admirals, captains, commanders, and lieutenants because it was a headquarters and a supply depot area.

**Gitschier:** That must have been a great experience, in terms of being a clinician.

**Smith:** That was real medicine! I mean I did physical examinations. If people got sick they came in to see me, and I would treat them. It was a very good two years. The plan up to that point was to continue on in medicine and become a practicing physician. But maybe an academic position, because I had the idea of wanting to do some research.

**Gitschier:** How did that idea of doing research get started?

**Smith:** From birth, I guess. [Laughter] I've always had kind of an inquiring mind. I always wanted to know how things worked.

I was first introduced to molecular biology while I was here in the Navy. And that laid the seeds for my eventually getting out of medicine and going into research.

**Gitschier:** How was that?

**Smith:** I had one afternoon a week free. And I could go up to the Naval Hospital. So I went up to the endocrine clinic, and worked there on, I think, Wednesday afternoons. And saw people with various disorders, among them, some genetic-type defects. And I started reading up on that. Somebody found that if you put the cells on a slide and then mashed the coverslip with your thumb, the cells would spread out and you could count the chromosomes. It was called a “squash prep,” and if you stained with orcein, you could very clearly see and count the chromosomes.

I read an article saying that there were 46 chromosomes, and then they started describing abnormalities in the chromosomes, like Turner syndrome, which is XO, and Klinefelter, which is XXY. And those people were coming into the endocrine clinic, so I started reading up on that, and I turned to basic genetics texts. And there it was—Watson and Crick. 1957 was when I found out about it, in a general college text by Dobzhansky. And there was a just a little bit on it.

So, I got very interested because I could see the implications of it. And there were no textbooks on molecular biology. I don't know if the term was even invented at that point. But there were two paperback compilations of papers. One was *Bacterial Genetics* by Adelberg and the other was Stent's book on bacterial viruses. So if you read those, you were on the cutting edge!

It was when I started my medical residency at Ford Hospital in Detroit that I would go to the library after eating lunch and sit in an easy chair. I was reading Mark Adams's book *Bacteriophages* that came out at that time and *The Chemical Basis of Heredity*, which came out in 1957. I was really very interested in this now, but I hadn't consciously made the decision to leave medicine at that point.

**Gitschier:** Because you still have a number of years of residency to go!

**Smith:** Right. So here I am in the second year of my residency and I had a very good friend, Pierre Caron, he was a French guy from Montreal. And he was kind of interested in science as well. And we had read about the Barr bodies, by which you could very easily determine male or female. So, we decided we'd play with that.

I can still remember we were making a stock solution of orcein. It was in the hood; we were heating up the acetic acid in a flask. And he says, “OK, I'm going to add the orcein,” and he reached in and put it in, and it went “Poof!” ‘cause we didn't have boiling chips, and we had super-heated the solution, and the whole hood was covered with purple stain.

Anyway, we got our stock solution and we started getting some buccal smears and started looking at Barr bodies. We were rotating through endocrinology at that point and there were some people with a question of whether they had a chromosomal defect or not. And the doctors in the clinic didn't know about this stuff, so we kind of introduced it.

Then I ran into Caron later on in the second year, and he said that he had applied for an NIH [National Institutes of Health] fellowship and he was going to take a two-year research fellowship before practicing. And so I thought, “Gee, that sounds good!” Sputnik went up in '57, so money was flooding into the system.

So I called my father and told him what I was planning to do. And he said, “Well there is this fellow Jim Neel at Ann Arbor [at the University of Michigan],” which is about 40 miles away. “Why don't you drive over and talk to him?”

**Gitschier:** How did he know Jim Neel?

**Smith:** Interesting story. My father [who was a professor in education at the University of Illinois] was up giving a lecture; and in the cafeteria line, Jim Neel was just ahead of him. And they struck up a conversation. Jim Neel had started that Department [of Human Genetics].

And so, I drove over and talked with him. He was very interested in me right away because of my mathematical background. He was doing statistical and genetic studies on isolated populations in the Amazon.

I immediately insulted him, because he said, “Would you be interested in doing this?” And I said, “No, I want to do basic research.” And he said, “Well, that's what I think I'm doing!” [Laughter]

**Gitschier:** But you meant something closer to the DNA.

**Smith:** I meant with the DNA and genes and so on. So when he heard what I was interested in—phage genetics and bacterial genetics—he said he had just hired Mike Levine, and maybe I could work with him.

Mike was at Brookhaven at the time. He was working on the *Salmonella* phage P22. So I wrote a very brief NIH Fellowship application. If you had reasonable credentials, it was no big deal [to get one].

**Gitschier:** Were you still thinking, like your friend Caron, “Well, after my fellowship, I'll then practice medicine”?

**Smith:** No, never again. Not once I got into the lab and saw that I understood and had a lot of ideas and so on.

So, [after two years] I had a paper in *PNAS* [*Proceedings of the National Academy of Sciences*] and then one in *Science* on temperature-sensitive mutations of the C genes, and Mike and I were getting along really well. Mike offered to make me a research associate, which is sort of a junior faculty position. So I was very happy. I was paid something like $9,000 a year. I moved over [from Detroit], bought a house, still not even thinking about the future. Probably Mike was worried—he didn't say anything though. I continued working for three [more] years. I think it was about the second year—about 1965—that he suggested that maybe I should start looking for a faculty position.

And then I got a call from Hopkins [saying] that they would like me to come out and give a talk.

**Gitschier:** Do you remember who at Hopkins called you?

**Smith:** Yeah, Dan Nathans.

**Gitschier:** Ah! Had you never met him before?

**Smith:** No, but the minute I visited there and gave the seminar, I knew that was it.

**Gitschier:** OK, so, this is something like 1967—and you are off to Hopkins. Tell me what you are working on once you got there and how you made this completely wild discovery.

**Smith:** Right. I had become very interested in lysogeny: the mechanism of how it's established, how when the phage genome enters the cell, it has to make a decision whether it's going to replicate itself or integrate into the genome to make a lysogen.

While still at Michigan I had discovered the *int* gene in *Salmonella* phage. And I wanted to get into biochemistry. So I decided I wanted to try to get an in vitro integration reaction. There was some early stuff starting to happen with lambda phage [which is very similar to P22]. There were a large number of lambda people. Like 50 of them, I think.

**Gitschier:** P22 was not as popular.

**Smith:** There were about two or three labs. I started out with a grant to study the integration process. I guess it was just after a few months that I got to Hopkins with my grant and everything, that I realized that the lambda people—there were too many of them! They were moving in on this area. And I didn't want to just do some…

**Gitschier:** A “me-too” experiment.

**Smith:** Yeah, exactly. So I decided I would start looking at general recombination. And I decided maybe if I had a transformation system, where I could do experiments in the test tube with DNA and then test to see if there was recombination by transforming, to see if markers were linked, or not linked—it was kind of a naïve thing—but …

It turned out at Hopkins there was Roger Herriott—just across the street at the School of Hygiene [and Public Health]. He had been studying *Haemophilus influenza* transformation for about a dozen years.

**Gitschier:** So that's how you got into the *Haemophilus*!

**Smith:** Yeah. So I spent a couple days working with his technician and learning how to grow and develop competent cells and do the assays.

**Gitschier:** And you couldn't do this in *Salmonella* [the P22 host]; they don't have natural transformation?

**Smith:** No, there is no transformation there. There is no genetically determined membrane system for taking up DNA. Whereas with *Haemophilus* or with *pneumococcus*, they all have genetically determined, very efficient uptake systems for DNA. And that's controlled by a couple dozen genes.

**Gitschier:** So the idea is you're going to make a recombinant in a test tube…

**Smith:** Right. I would take extracts of *Haemophilus*, and then treat two different DNAs carrying genetic markers and see if I could recombine them in the test tube, and then transform to show that I had double mutants.

I started doing some simple biochemistry experiments, and I was joined by a graduate student, Kent Wilcox. Just to get him going, I suggested that he take some phage P22 DNA that was labeled with ^3^H or ^32^P and that I had in the refrigerator. I said, “Take this DNA and transform it into *Haemophilus*” to see what happens. The idea was to let it go in and then to recover it from the cell to see what had happened to the DNA when it went in.

And we were also doing experiments with labeled *Haemophilus* DNA at the same time. And the cells would take it up and you could recover it again. But when he did it with the P22, nothing was recovered.

And here's where good fortune comes in. Matt Meselson and Robert Yuan had written a paper describing the first Type I restriction enzyme [Type I enzymes cut at a random distance from their recognition sites], and I gave a talk on it in the journal club, because it was a really fantastic paper. For the first time I really understood clearly what restriction and modification were. It was very clear now that there was an enzyme—an endonuclease—that recognized sites and cleaved the DNA.

**Gitschier:** And had you given this journal club just as Wilcox was doing this experiment?

**Smith:** I gave this a week before the initial idea came to us. I gave the seminar, he did his experiment, and he couldn't recover the foreign DNA from the cell. And he said, “Could it be restriction?”

**Gitschier:** Oh, he said that?

**Smith:** Yeah, he said that! And I said, “No!” And I was thinking, obviously he bummed up the experiment in some way!

**Gitschier:** But the control worked—he did recover the *Haemophilus* DNA.

**Smith:** Yeah, the control worked.

**Gitschier:** So he didn't bum up that part of the experiment.

**Smith:** [Laughter] Anyway, it turned out in subsequent years that is was *not* restriction. He couldn't recover it because it wasn't taken up.

**Gitschier:** Are you kidding?

**Smith:** I'm not kidding. The foreign DNA was not taken up because it didn't have uptake sites on it.

But I went home that night, and thought about it, and realized that we had a very simple assay that we would know in ten minutes what the answer was.

So, we came in the next morning. What I had realized was that I had been doing some work with viscometry, using *Haemophilus* extracts [which were sonicated to destroy their own DNA], to see if the DNA was getting broken down when you add the extract, or maybe it was getting put together and becoming more viscous. I was just playing around, basically. And I had already learned that [with] the *Haemophilus* DNA—nothing happened. You add extract, the viscosity would stay the same, just flat.

**Gitschier:** Tell me a little more about the experiment.

**Smith:** The chamber where the reaction is taking place has extract and buffer, magnesium, and so on. And then we added the *Haemophilus* DNA to it and mix it. And as you draw it back and forth in the capillary tube, the time it takes to go through the little hole didn't change. In other words, the viscosity [was the same]. The molecules were not getting broken or anything.

I realized that if we had a restriction enzyme, it's an endonuclease. It would begin to break internally, and the viscosity would drop very rapidly. A single break and the viscosity would drop. So we set up two viscometers—one with *Haemophilus* DNA and one with the P22 DNA, added the extract, mixed, and then started taking [measurements] as fast as we could do it. By five minutes, the first point on the P22 was way down, whereas the *Haemophilus* was the same. We had something in there recognizing that it was a foreign DNA: restriction.

**Gitschier:** OK. Before we continue on that, why were you so sure that Wilcox's suggestion about the restriction enzyme had been wrong?

**Smith:** Well, I mean, it was just too much to expect, you know! ‘Cause we didn't know that there were widespread systems at that time. It [restriction] was in [*E.*] *coli*. The original Meselson enzyme was in *coli*.

**Gitschier:** Right, as was the Werner Arber stuff.

**Smith:** Yeah, and I thought maybe it was a special mechanism in those bacteria. I mean, I wasn't dogmatic about it. Obviously, I started thinking about it.

But the crucial thing was that we had a simple assay to determine if it was restriction. I thought if it really *is* a restriction enzyme, then with the viscometry, we would be able to detect a single break very quickly! If he hadn't made the observation, of course, we probably wouldn't have done the work. And also, if I hadn't read that paper and presented it at the journal club…we never would have thought of it.

**Gitschier:** Do you remember what date that was?

**Smith:** Yeah, I do! It was May 28th of 1968.

**Gitschier:** Wow!

Now, let's fast-forward. Sometime in the mid-'90s, you started to work with Craig Venter.

**Smith:** Yeah, sure. Of course the human genome project started officially around 1990, but there was already money appropriated as early as 1987. And people were applying for grants, and I got one of those early grants, an R01, just to size the *Haemophilus* genome using restriction fragments.

So, at that point Craig was not in genomics. It was not till '91 that he began to become prominent.

**Gitschier:** And that was because of the ESTs [expressed sequence tags]?

**Smith:** ESTs, yes, and the first paper was published in '91. And that aroused a lot of academic anger, because of the patent issue. So that's when I first heard about Craig; he was in the center of a big controversy.

It was not until the spring of '93 that we both happened to go to a meeting in Bilbao, Spain. The meeting was convened to discuss legal, religious, and scientific issues related to the human genome project.

I was chairing one of the sessions in the meeting, actually, and Craig spoke in that. At the end of the day, I had gone over the hotel and into the bar just to relax a little bit, and Craig walks in and orders a drink. We started talking about how we got into science and there were some similarities.

He was a very congenial guy. I think when I first met him, I said something to the effect of “Where are your horns, because in academia you are the devil!” [Laughter]

**Gitschier:** And what did he say?

**Smith:** He didn't say anything—he just sort of grinned. We really hit it off well, and [later] he asked would I be interested in being on his scientific advisory council for his new Institute, which had just started about six months before that. So, I said, “I'll drive down and take a look.”

**Gitschier:** And this is TIGR.

**Smith:** This is TIGR. He had left NIH at that point. He had money from Human Genome Sciences and set up his Institute. He had an up-and-running lab and he was doing EST sequencing.

So I went down and took a look and I was totally blown away. He had this huge room with 30 sequencers. He was cranking out something like 400,000 base pairs a day, of raw sequence. That was huge.

So, I joined the council. They had their first annual meeting at some river retreat in Maryland, and I went to that. We were sitting around and he was describing how they were finishing off the EST sequencing sometime in early '94.

And I suddenly, I think a light went on in my head, and I said, “*Haemophilus*! If they can do 400,000 a day, we can sequence it in a few weeks.” So I raised my hand, and I said, “You call yourself The Institute of Genomic Research, let's do a genome! How about *Haemophilus influenzae*?”

**Gitschier:** So, at that point, he didn't have an idea that he was going to go into sequencing the human genome?

**Smith:** No. That was not yet on the radar screen. Even sequencing a whole bacterium was barely a blip. And he was very interested, because they were winding down the other stuff and he wanted something else to do. So I said, “I'll make a library of the genome and maybe we can sequence it.”

But I was thinking—and everybody at that time was thinking—that you make a library, you map the pieces, and then you sequence each piece. And then put it all together. That's the way they were doing *coli*, and it took almost ten years.

I went back to my lab group and I said, “Lookit, we could get the sequence of *Haemophilus*. We have to make this library and make these pieces.” And my group—they've all got their own projects. And they're looking at me, and they said, “That'll take a year and we don't have a grant for it.” Blah blah. I got very upset. I stormed off to my office.

**Gitschier:** Really, were you angry?

**Smith:** I was really disappointed with the group—here's an opportunity of a lifetime, and they didn't want to jump on it. I walked into my office and started thinking about it.

And I kept thinking. And I thought that we don't have the money and the time to do all this mapping—we've got to do it another way. And Craig is doing random shot-gun of ESTs and then assembling them into full transcripts. Let's do it that way. Let's make a single library, sequence a few tens of thousands of fragments, and put them all together with a computer.

And so, I drove down and everybody got together. It was a group of about 40–50 people—technicians and everyone—it was a nice group in those days. So, I got up to the board and I showed a couple of slides of a table, showing how as you sequence 5,000 fragments, you had this amount of genome completed, 10,000 you had this…and so on. And said, if we do 40,000, we'll close all the gaps.

And Craig, of course, was very excited. Everybody I think realized this was the way to do it. But Craig says, “No, we don't want to sequence 40,000. We'll sequence about 25,000, because you may never be able to close the gap. What we'll do is to assemble the 25,000 and we'll close the gaps after that.” And that's the way we did it.

**Gitschier:** Now, at some point you just leave Johns Hopkins altogether and go to work—was it at TIGR?

**Smith:** Yeah, 1998, July 1.

**Gitschier:** So you had been at Hopkins 31 years. What prompted you to close your lab at Hopkins and move?

**Smith:** Well, the success of the *Haemophilus* and the idea that I could become free of grants. One month later, I went to Celera.

**Gitschier:** OK, because then Celera started up. And Celera's intent was to sequence the human genome.

**Smith:** Yeah, and they picked Craig to be the president of the company and get the thing done.

**Gitschier:** And Craig invited you to come, too.

**Smith:** Well, I sort of insisted! He likes to quote me as saying, “I don't think this is going to work, but I want to go with you.” [Laughter]

**Gitschier:** Really? You didn't think it was going to work?

**Smith:** I knew they could sequence. I wasn't sure they could put everything together. But I wanted to be part of it, so I said, “I'm coming with you.”

**Gitschier:** OK! So, it's very interesting to me, you and he seem to be very different kinds of people. Yet, you've followed him out here…

**Smith:** Well—how can I say it? I would rank him as a genius. Extremely good in several areas. He's a superb scientist. He can see much further ahead than I can. So he's really started several independent areas of research. Like the ocean sampling and things—huge things that nobody in academia would even consider doing. We're able to do things [at JCVI] that you couldn't get any government funding for. It's too off-the-wall! They are too big, too costly, and too indefinite. And he's really good at that kind of thing. He's just an incredibly fascinating guy. And, he's willing to pay me to work.

**Gitschier:** And you're working on amazing stuff. Now, synthetic biology…where do you view this as going?

**Smith:** I fully expect that in a few years—I don't want to say in how many years—we'll be able to design new bacteria. We have to learn a lot before we can do that. We can already synthesize genomes. So that—the technical part of it—is there. The problem now is to know them well-enough to design organisms, and then we should be able to make them do things that we want them to do.

I'm only interested in the science. I want to learn enough about the essential genes in cells, because if you're going to design an organism, you have to start with an essential set of genes that make it alive. Then you can just add stuff onto it. So then we can have modules that make bio-fuels or a pharmaceutical product. Just plug it in. It's naïve at this point. But the future of actually designing bacteria.

**Gitschier:** And here you are back in San Diego. It sounds to me that you've got the world's greatest job!

**Smith:** Yeah, Craig is the guy that does everything. I'm just happy that I can continue to work. I mean I just turned 80 this year.

**Gitschier:** It's just fantastic! It's inspirational, really. I'm wondering what kind of advice you might have for scientists starting out today, given that you've had this long and varied career.

**Smith:** Everybody's different. It's hard to advise anybody. But, do stuff you like to do. That's very important for motivation. You should work on your own ideas, not other peoples' ideas, if you can. It's not just a job to make money.

